# Detecting taxonomic signal in an under-utilised character system: geometric morphometrics of the forcipular coxae of Scutigeromorpha (Chilopoda)

**DOI:** 10.3897/zookeys.156.1997

**Published:** 2011-12-20

**Authors:** Beatriz Lopez Gutierrez, Norman MacLeod, Gregory D. Edgecombe

**Affiliations:** 1 Department of Biological Sciences, Imperial College, London SW7 2AZ, UK; 2 Department of Palaeontology, The Natural History Museum, Cromwell Road, London SW7 5BD, UK

**Keywords:** Centipedes, forcipules, eigenshape, Procrustes superposition, canonical variates analysis

## Abstract

To date, the forcipules have played almost no role in determining the systematics of scutigeromorph centipedes though in his 1974 review of taxonomic characters Markus Würmli suggested some potentially informative variation might be found in these structures. Geometric morphometric analyses were used to evaluate Würmli’s suggestion, specifically to determine whether the shape of the forcipular coxa contains information useful for diagnosing species. The geometry of the coxae of eight species from the genera *Sphendononema*, *Scutigera*, *Dendrothereua*, *Thereuonema*, *Thereuopoda*, *Thereuopodina*, *Allothereua* and *Parascutigera* was characterised using a combination of landmark- and semi-landmark-based sampling methods to summarize group-specific morphological variation. Canonical variates analysis of shape data characterizing the forcipular coxae indicates that these structures differ significantly between taxa at various systematic levels. Models calculated for the canonical variates space facilitate identification of the main shape differences between genera, including overall length/width, curvature of the external coxal margin, and the extent to which the coxofemoral condyle projects laterally. Jackknifed discriminant function analysis demonstrates that forcipular coxal training-set specimens were assigned to correct species in 61% of cases on average, the most accurate assignments being those of *Parascutigera* (*Parascutigera guttata*) and *Thereuonema* (*Thereuonema microstoma*). The geographically widespread species *Thereuopoda longicornis*, *Sphendononema guildingii*, *Scutigera coleoptrata*, and *Dendrothereua linceci* exhibit the least diagnostic coxae in our dataset. *Thereuopoda longicornis* populations sampled from different parts of East and Southeast Asia were significantly discriminated from each other, suggesting that, in this case, extensive synonymy may be obscuring diagnosable inter-species coxal shape differences.

## Introduction

The Scutigeromorpha (Chilopoda) is the only extant representative of the centipede subclass Notostigmophora. These centipedes retain several primitive characters such as compound eyes, a domed head capsule, and deposition of the spermatophore on the ground rather than on a web. These characteristics, together with molecular sequence data, identify them as the sister group of all other centipedes (Murienne et al. 2010).

Scutigeromorph taxonomy in its present form was largely established by K. W. Verhoeff in a series of studies that spanned the first half of the 20^th^ century. Verhoeff (1905) was also the first investigator to present a hypothesis of phylogenetic relationships for the group. He named most of the genera (Verhoeff 1904, 1905, 1925, 1944) and a large number of species, many of which have been synonymised subsequently (see Würmli 1973a,b, 1977, 1978, 1979, 2005). Despite the inclusion of new morphological character data (e.g., from scanning electron microscopy) and extensive molecular sequencing (Edgecombe and Giribet 2006, 2009), the taxonomy and phylogenetic relationships of the ca 100 valid scutigeromorph species remain controversial, in part because many aspects of this group’s morphology are highly conserved.

**Figure 1.**
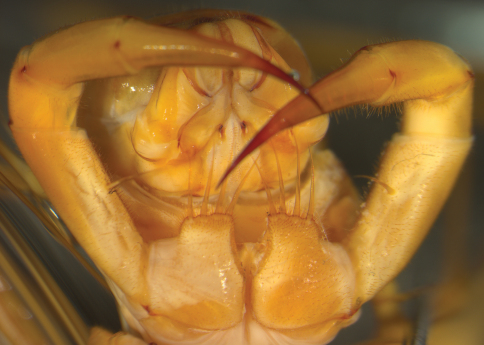
Ventral view of head and forcipules of *Thereuopoda longicornis* placed in a standard horizontal position. BM 1952.9.8.574-575, Kuching, Sarawak, Malaysia.

Among such complex, but apparently conservative, character systems are the forcipules, the appendages of the first trunk segment that are a functional part of the head and house the poison gland. Scutigeromorph forcipular coxae are separated from a vestigial sternite (Manton 1965) and each coxa bears four long spine-bristles along its anterior margin (Fig. 1). Würmli (1974) drew attention to the importance of the shape – the relative lengths and widths of the coxa — and the prominences on the inner margin of these structures. However, the degree to which these shape characters can be used to identify taxa (either species or infra/supraspecific groups) reliably has never been subjected to systematic investigation. Indeed, the forcipules have played almost no role in scutigeromorph systematics to date.

Geometric morphometrics has been used for the past 25 years to quantify biological form via the use of landmark and semi-landmark data (Rohlf and Marcus 1993, Adams et al. 2004, MacLeod 2002a, 2002b, 2005; see also Bolton et al. 2009 for an application to the female gonopods of Scutigeromorpha). Whereas a number of authors have suggested that morphometrics could be useful in resolving traditional taxonomic characters and contributing to taxonomic and phylogenetic analysis (e.g., MacLeod 2002a), morphometric approaches have traditionally been used to evaluate characters that have been recognized by taxonomists via qualitative inspection. In the case of the scutigeromorph forcipules, despite their morphological complexity these structures have defeated qualitative analysis; taxonomically and phylogenetically informative morphological characters have necessarily been sourced from other parts of the body.

The primary objective of this investigation was to determine the degree which the forcipular coxae can be used to characterise scutigeromorph taxonomic and phylogenetic groups accurately based on an assessment of their shape. In doing so, we also explored the extent to which Procrustes principal component analysis (see MacLeod 2010) as well as canonical variates analysis (CVA) and shape models calculated for the CVA ordination space can help identify characters that add support for existing or alternative taxonomic placements.

## Methods

### Taxonomic sampling

Specimens fixed in ethanol were sourced from The Natural History Museum (London). Specimens of the three Australian species were sourced from the Australian Museum (Sydney) and the Queensland Museum (Brisbane); specimens of two Dominican Republic species were located in the collections of the U.S. National Museum of Natural History.

Landmark and outline data were collected for 108 specimens (Table 1), representing eight species and eight genera within two of the three scutigeromorph families. The Neotropical/Afrotropical family Pselliodidae is here represented by *Sphendononema guildingii* (Newport, 1844), and the family Scutigeridae, where sampling was densest, includes members of both recognized subfamilies, Scutigerinae and Thereuoneminae. The Scutigerinae were represented by *Scutigera coleoptrata* (Linnaeus, 1758) and *Dendrothereua linceci* (Wood, 1867); the Thereuoneminae by *Thereuonema microstoma* (Meinert, 1886), *Thereuopoda longicornis* (Fabricius, 1793), *Thereuopodina queenslandica* Verhoeff, 1925, *Allothereua maculata* (Newport, 1844), and *Parascutigera guttata* Verhoeff, 1904. All species were chosen on the basis of being members of accessible collections with sample size adequate for statistical analysis, and to represent a broad sample of generic/subfamilial diversity. The remaining scutigeromorph family, Scutigerinidae, was not included as too few specimens were available. Voucher details for all specimens used in this study are listed in the Appendix (Table 1 therein).

**Table 1.** Sample number and distribution of species employed in the present study

**Species**	**Sample number (N)**	**Distribution**
*Sphendononema guildingii*	5	Central and South America
*Scutigera coleoptrata*	12	Mediterranean, cosmopolitan by introduction
*Dendrothereua linceci*	13	North-Central America, Caribbean
*Thereuonema microstoma*	14	East Africa, Middle East
*Thereuopoda longicornis*	19	India, southeast Asia
*Thereuopodina queenslandica*	14	Northeastern Australia
*Allothereua maculata*	13	Southern Australia
*Parascutigera guttata*	18	Northeastern Australia
**Total**	**108**	

The species concept for *Dendrothereua linceci* follows Würmli (1973b), applying this name to populations distributed from the southern U.S. to Panamá. Analyses of molecular data suggest that multiple species may be represented in this aggregation (Edgecombe and Giribet 2009), but using the traditional concept of a widespread species allows exploring the variability in this taxonomic grouping. Identifications of other widespread species follow their most recent revisions (*Thereuonema microstoma* = *Thereuonema syriaca*: Würmli 1975; Stoev and Geoffroy 2004; *Scutigera coleoptrata*: Würmli 1977; *Sphendononema guildingii*: Würmli 1978; *Thereuopoda longicornis*: Würmli 1979; *Parascutigera guttata*: Edgecombe and Giribet 2009). As in the study of Bolton et al. (2009), the name *Allothereua maculata* is applied to populations from arid parts of New South Wales and South Australia that belong to *Allothereua maculata* (sensu Verhoeff 1925), though their conspecificity with the Western Australian type material is dubious.

Specimens were chosen so that at least one of the forcipular coxae and the spine bristles on its anterior margin were visible and complete. To minimise the effects of variation due to potential shape changes in ontogeny, specimens are all mature or nearly mature (maturus and pseudomaturus stages of Verhoeff 1904) apart from some of the smaller *Dendrothereua linceci*.

### Image capture and landmarks

In order to quantify the outline of each forcipular coxa, specimens were placed ventral surface uppermost and pinned to a horizontal standard orientation (i.e., the forcipular coxae positioned as horizontal as possible) (Fig. 1). Each specimen was imaged perpendicularly using a Leica MZ16 stereomicroscope with a Zeiss Axiocam™ high-resolution imaging system.

Imaging the specimens in the correct orientation while in alcohol proved difficult as many specimens were not perfectly straight and even the slightest movement disturbed the alcohol and blurred the image. Additionally, the forcipular coxae are not completely flat, rendering some areas of the coxa out of focus. To overcome focusing problems, a stack of images of each specimen at different focal depths was taken and the set then merged to form a single, extended focus composite using HELICON FOCUS™ (Helicon Software Ltd.) software. All composite images were then cropped, placed on a black background, and contrast adjusted using Adobe PHOTOSHOP™ software while referring to the original images for guidance. Images were excluded if (1) the original image stack was insufficiently focused to accurately detect the coxal outline and/or the base of all the spine-bristles, and/or (2) their orientation did not conform to an acceptable standard.

**Figure 2.**
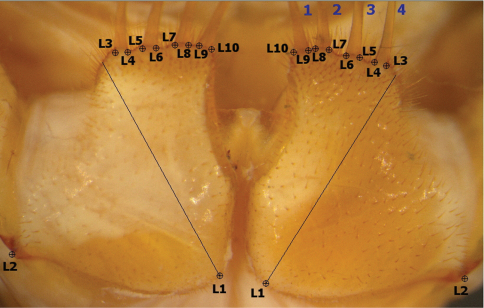
Landmarks (L1-L10) used in morphometric analysis. Diagonal line to L1 is the longest line from anterolateral to posteromedial corners of the coxa. Spine-bristles numbered 1-4 (blue) from interior to exterior. Throughout text, left and right coxae refer to dorsal orientation (inverted 180° relative to this ventral view).

Media Cybernetics’ IMAGE-PRO PLUS™ software was used to collect landmarks and outline co-ordinate points from the right and the left coxae individually. Symmetry between the right and left coxae was established (see Results section), and subsequently the left coxal landmark and outline coordinate points were reflected across the *y*-axis, only one coxa being used per specimen to eliminate redundancy. Ten landmarks (Fig. 2) were manually located from the interior to the exterior part of each coxa. The first landmark (L1) was calculated by drawing the longest diagonal line from the anterolateral to the posteromedial edge of the coxa, the second landmark (L2) was placed at the coxofemoral condyle, and landmarks L3-L10 were situated at the base of the projection bearing each spine-bristle. In addition, 100 equally-spaced outline semi-landmark co-ordinate points were obtained by automatic tracing along the mesial, posterior and lateral edges of the image from landmarks L10 to L3. The outline along the anterior margin (from landmarks 3 to 10) was obtained manually as the spine-bristles were not used in the shape analysis. These structures are very fragile and were disarticulated in a large subset of the specimens available or their length was partially obscured by other structures. Accordingly the manual tracing truncated the spine-bristles across their level of insertion into the coxae.

### Shape

Initially, combinations of both landmark and outline data were analysed using routines written in Wolfram Research’s MATHEMATICA*™* software using the semi-landmark sampling protocol described by MacLeod (1999). This protocol, part of the extended eigenshape analysis procedure, combines the coordinates of semi-landmark points along the outline and landmarks placed at comparable geometrical points which constrain the sequencing of the boundary coordinate points by forcing them into alignment, thereby reducing the degree of shape variation generated through biological miscorrespondence (MacLeod 1999). For this investigation a shape accuracy tolerance criterion of 0.975 was used to control the outline interpolation process. The analysis generated a series of *x-y* boundary outline (= semi-landmark) coordinate values that were used to represent the outline shape of each coxa.

No attempt was made to ‘slide’ the resulting semi-landmark points to positions of minimum bending energy relative to the sample mean as has been advocated for use in the analysis of semi-landmark data by some (e.g., Bookstein 1996, Zelditch et al. 2004). Such a transformation would destroy the shape correspondences that are the point of morphometric analysis. Moreover, all current implementations of the sliding semi-landmark approach of which we are aware do not slide the semi-landmark along the boundary outline itself, but rather along straight lines tangent to the boundary outline (see Rohlf’s documentation for the tpsRelw program, available as part of that program at the SB Morphometrics web site: http://life.bio.sunysb.edu/morph/). This convention has been adopted to simplify the computations required to reposition the semi-landmarks. However, use of this approach to semi-landmark adjustment obviously does not achieve a configuration of minimum bending energy for the curve that was measured because the act of sliding semi-landmarks along local tangents deforms the original measured curve. This wholly artificial deformation will not apply uniformly to the entire shape, but will be more pronounced in regions that exhibit high outline curvatures. Irrespective of this consideration, experiments have shown that minimal semi-landmark sliding results from the high-density sampling program used in extended eigenshape analysis.

The output of semilandmark interpolation procedure was aligned using Procrustes (GLS) superposition, which minimizes differences in position, scale, and rotation for sets of landmarks and semilandmark points (MacLeod 2009a). The superimposed coordinates were then analysed via PCA using the covariance matrix as a basis for the assessment of shape similarity. This technique explores the relations between variables to create new independent variables, the set of principal components, which represent a variance-optimised and mutually independent set of shape descriptors derived from the information contained in the original measurement set, reducing the dimensionality of the data set (Dunteman 1989; MacLeod 2005). In addition, this set of variables (= eigenvectors) can be used to define an ordination space that can be used to graphically portray shape relations and to create models of shape deformation trends that graphically embody the geometric meanings of the variables (Bookstein 1991; MacLeod 2009b, 2010).

Subsequently, canonical variates analysis (CVA) was performed on the PCA scores on a shape variance-optimised subset of the PCA axes to maximize the ratio of between-group and within-group variation for the eight species, thus discriminating between the groups (Zelditch et al. 2004). CVA was also used to investigate asymmetry differences between the right and left coxae, sexual dimorphism, and whether these shapes are informative for geographical patterns at an infraspecific level.

The geometric interpretation of the CVA axes that support group separation was then assessed using a CVA modelling procedure that projects points from the CVA space into the original PCA variable space to calculate a series of shape models that express the major shape trends involved in inter-group separation (see MacLeod 2007; Bolton et al. 2009).

## Results

In order to determine whether left and right coxae exhibit shape differences, data collected from both sides of specimens were subjected to CVA after coordinate data from the right coxae were reflected across the *y*-axis. No obvious differences in the scatter of left and right coxa along the first discriminant axes were evident. This result was then confirmed statistically using a likelihood ratio test (see Manley 1994; ф = 9.66, df = 12, p > 0.001). This same procedure was used to test for significant male-female shape differences (ф = 41.11, df = 24, p > 0.001). In both cases the null hypotheses of no between group shape differences could not be rejected. Additionally, a measurement error analysis was carried out using two randomly selected replicates of 12 specimens to estimate the level of data-collection accuracy that was achieved for the data set as a whole. This analysis also identified no significant statistical coxal shape differences (ф = 0.385, df = 9, p > 0.001) between replicate data collection sessions.

**Figure 3.**
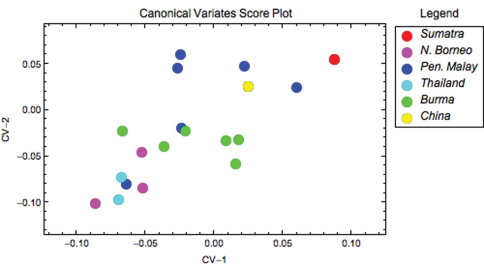
*Thereuopoda longicornis* scatterplot of coxal shape data along the discriminant subspace formed by the first two CV axes, which together account for 74.17% of observed between-group shape variation.

Ordinations of coxal outline shape projected along the first few Procrustes PCA axes for each species analysed independently did not reflect any obvious infraspecific clusters. The structure of infraspecific coxal shape was further investigated by grouping the data within *Thereuopoda longicornis* and *Dendrothereua linceci* by geographic locality. These geographically widespread species were selected because sufficient specimens were available from enough localities to allow a more rigorous comparison. Results of a CVA of the Procrustes PCA score data rejected the null hypothesis of no significant infraspecific differences in coxal shape between geographic groups in *Thereuopoda longicornis* (likelihood ratio: ф = 71.14, df = 22, p < 0.001). This result was supported by unexpectedly impressive discriminations by the CVA results. The set of CVA discriminant function axes revealed that specimens of *Thereuopoda longicornis* were assigned to their correct geographic group with 89% accuracy (Table 2), indicating marked and consistent infraspecific coxal shape differences. Furthermore, the inspection of CVA scatterplots (Fig. 3) shows that specimens from each of six pre-defined geographic regions within the species’ ordinations plot close to each other in the discriminant space. Results for *Dendrothereua linceci* are shown in the Appendix (Fig. 1).

**Table 2.** CV discriminant function analysis of *Thereuopoda longicornis* geographic data.

**Groups**	**Sumatra**	**N. Borneo**	**Peninsular Malaysia**	**Thailand**	**Burma**	**China**	**Total Correct**	**% Correct**
**Sumatra**	1	0	0	0	0	0	1	100
**N. Borneo (Sarawak)**	0	2	0	0	1	0	2	67
**Peninsular Malaysia**	0	1	5	0	0	0	5	83
**Thailand**	0	0	0	2	0	0	2	100
**Burma**	0	0	0	0	6	0	6	100
**China**	0	0	0	0	0	1	1	100
						**Total Correct**	**17**	**89**

**Figure 4.**
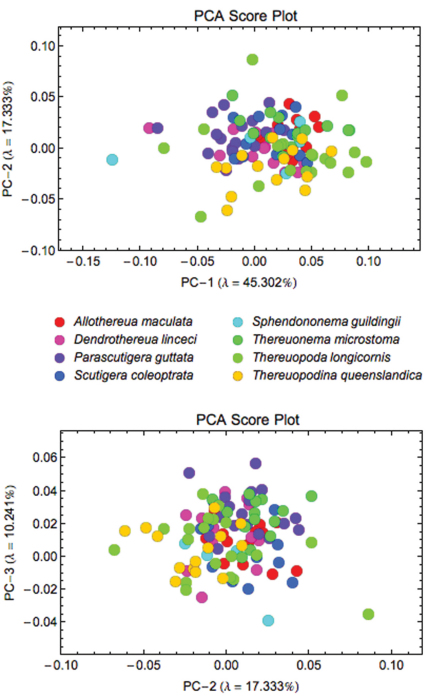
Scatterplots of Procrustes PCA scores for coxal shape data. The first two shape variation axes (top) together account for 62.63% of the observed shape variation; PC-2 and PC-3 axes (bottom) together account for 27.58% of the observed shape variation.

**Figure 5.**
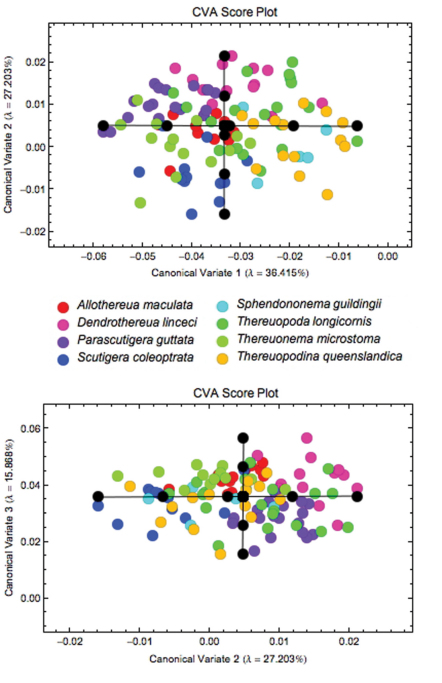
Results of the CVA of coxal shape data for all eight species, showing the subspaces formed by the first three discriminant axes, which together account for more than 79% of observed between-group shape variation. Within each subspace plot the black circles represent the coordinate locations for each of the five along-axis shape models depicted in Fig. 6.

When the sample of all eight species was pooled, Procrustes PCA identified a total of 12 axes that were required to account for 95% of the observed coxal shape variation (Fig. 4). Subsequent CVA analysis of the Procrustes PCA scores for these 12 latent shape variables revealed a clear tendency to group separation that is more pronounced for some species (*Thereuopodina queenslandica*, *Parascutigera guttata*, *Thereuonema microstoma* and *Allothereua maculata*) than for others (*Thereuopoda longicornis* and *Sphendononema guildingii*; see Fig. 5). There is a high degree of overlap evident in plots of species scatter in low-dimensional CVA subspaces. However, the degree of this true overlap among species point clouds is exaggerated in such plots as only two or three (out of the seven) discriminant axes were used in constructing the figure. Using the complete set of CV discrimination function axes, the overall proportion of correct specimen assignments (77%) was unexpectedly high for a character complex previously regarded as being of little taxonomic value. This, along with the likelihood ratio test results (ф = 308.5, df = 84, p < 0.001), indicates the presence of substantial and consistent between-species coxal shape differences for these data. *Parascutigera guttata*, *Allothereua maculata* and *Thereuonema microstoma* exhibit the most distinct coxal shape with 94%, 85%, and 79%, respectively, of specimens correctly assigned based on discriminant function analysis, whereas the least distinct were *Sphendononema guildingii* (60%) and *Thereuopoda longicornis* (68%). A jackknifed identification test to assess the stability of the discriminant axes (Table 4) indicates that, across the dataset as a whole, the individual training set outlines were assigned to the correct species with 61% accuracy. The most stable results, with reference to generalized group identification, are those for *Parascutigera guttata* and *Thereuonema microstoma* (89% and 71% correct identifications, respectively).

**Figure 6.**
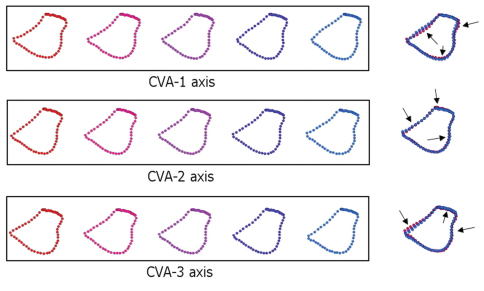
Strobe models of five positions along the canonical variates indicated in Fig. 5. CV-1, CV-2, and CV-3 axes account for 79.5% of the observed between-species shape variation. Landmarks and semi-landmarks are superimposed in the figure to the right of each sequence to express the magnitudes and directions (arrows) of shape trends. In all models, the mesial margin of the coxa is depicted to the left, the lateral margin to the right.

Figure 6 illustrates the forcipular coxal models that project three of seven CVA axes back into the space of the original Procrustes PCA axes. These three axes express more than 75% of between-species shape differences. The major shape trends illustrated in the first three CV axes were repeated along the four remaining axes. Models calculated for CV-1 show clear and pronounced variation from the coxofemoral condyle to the fourth spine-bristle (i.e., the course of the lateral margin of the coxa), which trends from distinctly concave to slightly convex, corresponding to a relative increase in coxal width. In addition, the posteromedial coxal edge also indicates a slight variation, further increasing the coxal width in its posterior part. In contrast, a small variation in the anterior medial margin of the coxa involves a reduction in the width of the coxa in its anterior part.

The CV-2 models also exhibited variation in the exterior/lateral margin of the coxa. However, variation here is (expectedly) more subtle than in CV-1 and the shift oriented in the opposite direction. The anterior margin of the coxa of CV-2 illustrates some variation anteriorly, particularly between the second and the fourth spine bristles, reducing the coxal length. In contrast, there is a slight variation in the posteromedial edge of the coxa that increases the coxal width in its posterior part.

The CV-3 model series identifies changes in three different areas of the coxa: (1) extension towards the coxofemoral condyle becomes more prominent; (2) the anterior edge exhibits a variation similar to CV-2 but in reverse, slightly increasing the coxal length; (3) the coxal interior margin exhibits a shift that reduces the coxal width. The overall coxal shape displays a slight increase in relative length and a reduction in width along the CV-3 axis.

Using the CV models from Figure 6 and the canonical variates space (Fig. 5) to interpret these results taxonomically, it can be seen that *Parascutigera guttata* has a relatively narrower forcipular coxa than the other sampled taxa, exhibiting a concave exterior margin with a very pronounced extension to the coxofemoral condyle. Alternatively, *Thereuopodina queenslandica* and *Dendrothereua linceci* display relatively wider coxae. *Thereuopodina*, however, exhibits a convex exterior margin, whereas *Dendrothereua* has a slightly shorter coxa with a concave exterior margin. In contrast, most specimens of *Thereuonema microstoma* and *Scutigera coleoptrata* exhibit a long and narrow coxa; *Thereuonema* exhibits a slight concave exterior margin and more pronounced coxofemoral condyle, while *Scutigera* has a straight exterior coxal margin. *Allothereua maculata* specimens are characterized by intermediate shapes along the axes. As reflected by their discriminant function results (Tables 3, 4), *Thereuopoda longicornis* and *Sphendononema guildingii* each display substantial variability in forcipular coxal shape.

## Discussion

The morphometric results obtained from the Procrustes PCA of these landmark-registered semi-landmark outline data and the CVA models back-projected in the PC space suggest that forcipular coxal shape differs significantly between taxa at various taxonomic levels. This character complex is clearly valuable in the assessment of scutigeromorph systematics because the group has historically been classified on the basis of a small number of taxonomically informative characters (principally exoskeletal prominences on the tergal plates and female gonopod shape). Our finding that specimens can for the most part be assigned to species with a high degree of accuracy (Table 3) substantiates a prediction by Würmli (1974) that coxal shape contains taxonomic information. Until now, however, this variation had not been quantified or harnessed taxonomically or phylogenetically. The outlines and landmarks illustrated in Figure 2 may possibly form the bases for future analysis of coxal differences.

**Table 3.** CV discriminant function analysis showing the percentage of specimens that were correctly assigned to their original species.

**Taxa**	*Allothereua maculata*	*Dendrothereua linceci*	*Parascutigera guttata*	*Scutigera coleoptrata*	*Sphendononema guildingii*	*Thereuopoda longicornis*	*Thereuonema microstoma*	*Thereuopodina queenslandica*	**Total Correct**	**% Correct**
*Allothereua maculata*	11	0	0	0	0	1	1	0	11	85
*Dendrothereua linceci*	2	9	2	0	0	0	0	0	9	69
*Parascutigera guttata*	0	0	17	0	0	1	0	0	17	94
*Scutigera coleoptrata*	1	0	0	9	0	0	2	0	9	75
*Sphendononema guildingii*	0	1	0	0	3	0	0	1	3	60
*Thereuopoda longicornis*	0	2	1	1	1	13	0	1	13	68
*Thereuonema microstoma*	1	0	1	0	0	0	11	1	11	79
*Thereuopodina queenslandica*	0	1	0	0	1	2	0	10	10	71
								**Total Correct**	**83**	**77**

Controversies over the status of particular species, and indeed the species concept applied across the group as a whole, are informed by the results of this study. For example, the geographically widespread *Thereuopoda longicornis* shows a variable coxal shape and, as a result, a relatively poor capacity for assigning specimens accurately, with 68% correct discrimination of the training set (Table 3). Furthermore, coxal shape variability in *Thereuopoda longicornis* has a discernible geographical pattern (Fig. 3), with specimens from each sampled southeast Asian region plotting near each other. Verhoeff (1905, 1937, and elsewhere) distinguished an array of nominal species of *Thereuopoda* that were later synonymised into *Thereuopoda longicornis* by Würmli (1979). This difference in the number of valid species (as extreme as 26 species versus one) reflects, at least in part, the observation that some characters employed at the species level by Verhoeff were subsequently found to have overlapping variation as sample sizes were increased, and in part reflects a historical shift towards species being seen as polymorphic, geographically widespread entities by later 20^th^ century taxonomists. The coxal shape differences observed between geographic groups of *Thereuopoda longicornis* (specimens assigned to their correct geographic group with 89% accuracy; see Table 2) suggest that some of the subjective synonyms of *Thereuopoda longicornis* may actually include valid species.

Another species with a comparably wide geographic range and complex taxonomic history, *Sphendononema guildingii*, likewise has a comparatively poor capacity to be discriminated (Tables 3, 4). The family Pselliodidae has long been identified as a high-ranking clade (Verhoeff 1904; Würmli 1978, 2005; Edgecombe and Giribet 2006). Our CVA models (Fig. 6) show that although *Sphendononema* has a relative short and wide forcipular coxa with a convex exterior margin, it is not readily distinguished from some members of Scutigeridae on coxal shape alone. Current taxonomic practice (including species identifications in this study) follows Würmli (1978) in placing many (22) nominal species in synonymy with *Sphendononema guildingii*, though some aspects of variation (e.g., female gonopod variability explored by Bolton et al. 2009) suggest the presence of multiple species. The low discriminant function scores for *Sphendononema guildingii*, in spite of a small sample size, would be consistent with a mixed-species sample.

Würmli (1973b) and Würmli and Negrea (1977) united *Scutigera linceci* as a single geographically widespread species which was later recognised as multiple species of *Dendrothereua* by Edgecombe and Giribet (2009). The latter regarded it as ‘exceedingly doubtful’ that *Dendrothereua linceci* constitutes a single species because specimens from the limits of the geographic range (Costa Rica versus the southern U.S.) had molecular sequence divergences that greatly exceeded those in uncontroversial morphospecies. Although no significant infraspecific coxal differences can be related to geography, pooling specimens from each of Mexico (Guerrero), Guatemala and Hispaniola (Appendix, Fig. 1 therein), both the raw and jackknifed discriminant function scores (69% and 46%, respectively) indicate that a relatively low percentage of specimens are correctly assigned to this species (Tables 3, 4). Coxal shape does not provide strong corroboration for *Dendrothereua linceci* being a single species.

The small sample size for some of the geographic groups within *Scutigera coleoptrata* prohibits statistical testing of whether infraspecific coxal shape differences can be related to geography. Würmli (1973a, 1977) revised the species-level taxonomy of *Scutigera*, proposing the synonymy of several species with *Scutigera coleoptrata*, consistent with the view that *Scutigera coleoptrata* is a synanthropic species throughout large extents of its geographic range, as well as with short molecular branch lengths between specimens from populations in different parts of that range (Edgecombe and Giribet 2009). The specimens studied here include native parts of the species’ distribution (Italy, Greece, Madeira, Algeria) as well as introduced parts (St. Helena, Bermuda). The assignment of specimens to this species with 75% accuracy for raw CVA scores (Table 3) does not appear to be a stable result based on the poor capacity for identification in CVA jackknife tests (Table 4).

**Table 4.** CV discriminant function of jackknife analysis showing the percentage of specimens that were correctly assigned to their original species.

**Taxa**	*Allothereua maculata*	*Dendrothereua linceci*	*Parascutigera guttata*	*Scutigera coleoptrata*	*Sphendononema guildingii*	*Thereuopoda longicornis*	*Thereuonema microstoma*	*Thereuopodina queenslandica*	**Total Correct**	**% Correct**
*Allothereua maculata*	9	0	0	1	0	1	2	0	9	69
*Dendrothereua linceci*	4	6	2	0	1	0	0	0	6	46
*Parascutigera guttata*	0	0	16	1	0	0	1	0	16	89
*Scutigera coleoptrata*	1	0	0	5	1	1	3	1	5	42
*Sphendononema guildingii*	0	0	1	0	2	0	0	2	2	40
*Thereuopoda longicornis*	1	2	1	1	1	11	1	1	11	58
*Thereuonema microstoma*	2	0	1	0	0	0	10	1	10	71
*Thereuopodina queenslandica*	0	1	0	0	2	2	2	6	6	46
								**Total Correct**	**65**	**61**

In contrast to the variability discerned in the species discussed above, some species, notably *Parascutigera guttata* and *Thereuonema microstoma*, display consistently distinct coxal shapes (Table 3) and these results are stable when subjected to a jackknife test (Table 4). The diagnosability of these species with respect to coxal shape is in agreement with recent classifications that have established *Parascutigera guttata* (Edgecombe and Giribet 2009) and *Thereuonema microstoma* (Stoev and Geoffroy 2004) as valid species. Both species have narrow forcipular coxae, but *Parascutigera guttata* has a pronounced concave exterior margin with a distinct projection of the coxofemoral condyle whereas *Thereuonema microstoma* exhibits a longer coxa with a straighter exterior margin. It should be noted that the high discriminant function score for *Thereuonema microstoma* might be expected to decrease were more specimens from distant parts of its geographic range included; nearly all specimens used in this study were from a small part of its total distribution, in the Sudan.

## Conclusion

Geometric morphometrics of forcipular coxal shape indicates that these structures contain taxonomic information at the species level. Discriminant function analysis indicates that a majority of specimens of all eight species sampled in this study were assigned correctly according to their established taxonomy, in several cases with a high degree of accuracy. This investigation also demonstrates that morphometric approaches and CVA modelling procedures can be of considerable use in the analysis of subtle morphological features, and can support a wide variety of comparisons between groups at different taxonomical levels even in character systems that had been opaque to qualitative analysis, such as the forcipular coxae of Scutigeromorpha.

## Appendix 1

Voucher data for specimens use din morphometric analyses and supplementary figures of Canonical Variates scatterplots. File format: Adobe Acrobat (pdf) file.
